# Natural Deep Eutectic Solvent (NaDES) Extraction, HPLC-DAD Analysis, and Antioxidant Activity of Chilean *Ugni molinae* Turcz. Fruits

**DOI:** 10.3390/antiox14101234

**Published:** 2025-10-14

**Authors:** Javier Antileo-Laurie, Verónica Olate-Olave, Valentina Fehrmann-Riquelme, Camila Anabalón-Alvarez, Luis Cid-Carrillo, Javier Campanini-Salinas, Carlos Fernández-Galleguillos, Luisa Quesada-Romero

**Affiliations:** 1 Escuela de Química y Farmacia, Facultad de Ciencias, Universidad San Sebastián, General Lagos 1163, Valdivia 5090000, Chile; jantileol@correo.uss.cl; 2 Departamento de Bioquímica Clínica e Inmunohematología, Universidad de Talca, Avenida Lircay s/n, Talca 3460000, Chile; volate@utalca.cl; 3 School of Nutrition and Dietetics, Faculty of Rehabilitation and Quality of Life Sciences, Universidad San Sebastían, General Lagos 1163, Valdivia 5090000, Chile; vfehrmannr@correo.uss.cl (V.F.-R.); canabalona1@correo.uss.cl (C.A.-A.); lcidc1@correo.uss.cl (L.C.-C.); 4 Escuela de Química y Farmacia, Facultad de Ciencias, Universidad San Sebastián, Lago Panguipulli 1390, Puerto Montt 5501842, Chile; javier.campanini@uss.cl; 5 Departamento Biomédico, Facultad de Ciencias de la Salud, Universidad de Antofagasta, Antofagasta 1270300, Chile

**Keywords:** polyphenols, murta, *Ugni molinae*, edible native fruits, antioxidant activity, NaDES, HPLC-DAD

## Abstract

The demand for sustainable methods to extract bioactive compounds from native fruits is increasing. We evaluated the potential of natural deep eutectic solvents (NaDES) combined with ultrasound-assisted extraction (UAE) to recover phenolic compounds from *Ugni molinae* Turcz. (murta), a Chilean fruit with recognized ethnopharmacological and antioxidant value. Seven choline chloride-based NaDESs (M1–M7) were assessed and compared with conventional methanol: formic acid extraction (M8). The choline chloride: 1,2-propanediol system (1:2, M2) achieved the highest recovery of total phenolics (64.87 mg GAE/g) and flavonoids (35.38 mg QE/g), together with strong antioxidant activity (DPPH IC_50_: 1.05 µg/mL; ORAC: 40,291 µmol TE/g). When comparing the different NaDES formulations (M1–M8), M8 displayed superior FRAP and ORAC values, although its phenolic and flavonoid yields were lower, reflecting differences in solvent selectivity. HPLC-DAD analysis further revealed that NaDES, particularly M5 (choline chloride: oxalic acid, 1:1), favored the extraction of flavonoid and anthocyanin-type compounds. Multivariate and PCA analyses showed distinct chemical profiles in NaDES extracts, forming two clusters apart from M8. Pearson correlation analysis linked antioxidant capacity with major flavonoids. Overall, NaDES combined with UAE represents an efficient, green strategy for selectively recovering bioactives, supporting applications in foods, nutraceuticals, and health products from Chilean native fruits.

## 1. Introduction

Edible wild fruits are a group of plant-based foods known for their nutraceutical properties, which have been widely studied in recent decades for their role in health promotion and disease prevention [1,2]. Among the bioactive constituents in edible fruits, polyphenols (including flavonoids, phenolic acids, tannins, and stilbenes) represent one of the most abundant and diverse groups of secondary phytochemicals. Polyphenols are characterized by hydroxyl groups on aromatic rings, which give them their remarkable antioxidant power. The ability of polyphenols to scavenge reactive oxygen species (ROS) is considered one of their principal antioxidant mechanisms [3]. ROS are highly unstable, and their excessive accumulation leads to oxidative stress, promoting cellular damage and tissue injury associated with ageing, atherosclerosis, carcinogenesis, and mutagenesis [4].

*Ugni molinae* (commonly known as murta) is a shrub of the Myrtaceae family, native to the central-southern region of Chile, that produces an edible fruit widely consumed by local communities for its anti-inflammatory, analgesic, and kidney-protective benefits [5]. The phytochemical composition of *U. molinae* is characterized by a high polyphenol content [6], mainly flavonols such as quercetin, rutin, kaempferol, luteolin and myricetin derivatives, along with phenolic acids, proanthocyanidins, and anthocyanins [7,8,9], whose levels may vary with geographic and climatic conditions. Additionally, its high antioxidant capacity, together with a broad spectrum of health-promoting activities [6,10,11,12,13], highlights the considerable biomedical and commercial potential of this fruit.

Several methods have been reported for the extraction of polyphenols from edible fruits, including *U. molinae*, each presenting specific advantages and limitations. In this context, natural deep eutectic solvents (NaDES) have emerged as a promising alternative, offering enhanced sustainability and extraction efficiency. NaDES are biodegradable, non-volatile, non-flammable, and cost-effective, positioning them as an environmentally friendly option in green chemistry [14,15].

They are prepared by combining naturally occurring compounds (such as lactic acid, sugars, or amino acids) that act as hydrogen bond donors (HBD) and acceptors (HBA), and have attracted considerable attention as sustainable alternatives to conventional solvents. NaDES have shown strong potential for polyphenol extraction, providing advantages over traditional methods such as improved solubility of target compounds, reduced extraction times, higher yields, and lower toxicity [15,16,17,18]. Nevertheless, their overall sustainability ultimately depends on the components employed.

Recent studies have also confirmed their applicability in food raw materials, including the recovery of polyphenols from pomegranate peels [19], anthocyanins from chokeberry fruits [20], and flavonoids from citrus pomace [21], highlighting their value in functional food and nutraceutical research. In this framework, combining NaDES with ultrasound-assisted extraction (UAE) emerges as a particularly promising strategy, offering not only a green and efficient approach but also strong selectivity for flavonoids, which facilitates purification and enhances antioxidant activity [22,23].

This study aimed to evaluate for the first time the antioxidant potential of polyphenols extracted from *Ugni molinae* fruits using a series of choline chloride-based Natural Deep Eutectic Solvents (NaDES) combined with Ultrasound-Assisted Extraction (UAE). The results were then compared with those obtained using conventional methanol: formic acid extraction. To compare, the chromatographic profiles of the extracts were obtained, and the extraction efficiency was assessed using High Performance Liquid Chromatography coupled to a Diode Array Detector (HPLC-DAD). Finally, multivariate statistical analyses, such as Principal Component Analysis (PCA), and correlation assays were performed to relate the polyphenolic profile to the total phenolic and flavonoid contents, as well as the measured antioxidant capacity.

## 2. Materials and Methods

### 2.1. Solvent and Reagents

Analytical-grade reagents, including acetate buffer, 2,4,6-tripyridyl-s-triazine (TPTZ), HCl, FeCl_3_, Folin–Ciocalteu reagent, acetone, sodium bicarbonate, 1,1-diphenyl-2-picrylhydrazyl (DPPH), Trolox (6-hydroxy-2,5,7,8-tetramethylchroman-2-carboxylic acid), fluorescein, AAPH (2,2′-azobis(2-methyl-propionamidine) dihydrochloride), iron chloride, choline chloride, glycerol, 1,2-propanediol, citric acid, lactic acid, oxalic acid, and urea were purchased from Sigma-Aldrich (St. Louis, MO, USA). Reference HPLC standards—gallic acid, rutin, quercetin, and cyanidin-3-*O*-glucoside (purity ≥ 99%)—were also obtained from Sigma-Aldrich. Acetonitrile and formic acid (chromatographic grade) and ultrapure water were obtained from a Millipore system (Billerica, MA, USA). For chromatographic analysis, samples and solvents were filtered using 0.22 μm and 0.45 μm membranes, respectively, both from Millipore.

### 2.2. Plant Material

Ripe fruits of *Ugni molinae* were manually harvested and obtained from the coastal area of Curiñanco, Región de Los Ríos, Chile (39°73′30.0″ S, 73°38′45″ W), during fall 2023 (Figure S1). The experimental field corresponds to a temperate rainforest climate with soils of high water retention and good infiltration capacity. After harvesting, the fruits were washed, selected, and cleaned at the Laboratory of Food and Bioactivity Compounds of Universidad San Sebastián, and then stored in an ultra-freezer at −80 °C until further processing.

### 2.3. Extraction Process

The fruits were lyophilized using a freeze dryer system (Biobase, BK-FD10P, Jinan, Shandong, China) at −45 °C for 72 h under a vacuum pressure of 0.1 mbar. Once lyophilization was completed, the fruits were ground with a food processor (Thomas Elektrogeräte, TH-8706, Shanghai, China) and sieved through a stainless-steel sieve with a 0.5 mm mesh until a fine, dry powder was obtained. The processed dried fruits were extracted using Natural Deep Eutectic Solvents (NaDES) (treatments M1 to M7) or with methanol:formic acid (95:5; *v*/*v*) as a traditional extraction method (control, M8). NaDES were prepared by mixing the components in glass vials (final volume: 20 mL each) at a molar ratio of 1:1 or 1:2, followed by the addition of 30% (*v*/*v*) of ultrapure water to each NaDES to reduce viscosity. Then, the mixtures were placed in a water bath at 80 °C for 1 h until a homogeneous phase formed. All NaDES included choline chloride as the hydrogen bond acceptor, combined with a polyalcohol or a carboxylic acid as the hydrogen bond donor at different molar ratios [16], as specified in Table 1.

#### 2.3.1. NaDES Extraction

For NaDES extraction, 1 g of freeze-dried fruit was added to glass vials containing 20 mL of each NaDES mixture and homogenized for one minute using an Ultra-Turrax homogenizer (Janke & Kunkel, Ika-Werk, SD-45, Staufen im Breisgau, Baden-Württemberg, Germany). Subsequently, the mixtures were placed in an ultrasonic bath (Elmasonic S, Elma Schmidbauer GmbH, Singen, Baden-Württemberg, Germany) and sonicated for 40 min (frequency: 50–60 Hz, temperature: <40 °C). All samples were then centrifuged (Zentrifuge CAT. DM0412, DLAB, Riverside, CA, USA) at 4500 rpm for 60 min. The resulting supernatants were vacuum-filtered to obtain clean, particle-free extracts. Extracts were stored at 4 °C until further analysis. All extractions were performed in triplicate.

#### 2.3.2. Methanolic Extraction

For methanolic extraction, a similar process was followed. Briefly, 1 g of freeze-dried fruit was added to glass vials containing 20 mL of a methanol:formic acid solution (95:5; *v*/*v*) in triplicate. The samples were homogenized for one minute using an Ultra-Turrax apparatus (Janke & Kunkel, Ika-Werk, SD-45, Staufen im Breisgau, Baden-Württemberg, Germany) and subsequently placed in an ultrasonic bath (Elmasonic S, Elma Schmidbauer GmbH, Singen, Baden-Württemberg, Germany) for 40 min (frequency: 50–60 Hz, temperature: <40 °C). The samples were then centrifuged (Zentrifuge CAT. DM0412, DLAB Scientific Inc., Riverside, CA, US) at 4500 rpm (60 min). The resulting supernatants were vacuum-filtered and concentrated using a rotary evaporator (DLAB Scientific Co., RE100 Pro, Beijing, China) at <40 °C to remove the organic solvent. Finally, the extracts were stored at 4 °C until further analysis.

### 2.4. Spectrophotometric Analysis

For each assay, spectrophotometric analyses were performed in triplicate, including a sample blank for baseline correction, and measured at the appropriate wavelengths using a multimode microplate reader (Synergy H1, BioTek Instruments, Winooski, VT, USA), as described below.

#### 2.4.1. Total Phenolic Content (TPC)

The antioxidant activity of the extracts was evaluated using several antioxidant assays, with modifications as described in our previous work [24]. Briefly, 15 µL of each obtained extract was placed in a microplate well and then, 165 µL of Folin–Ciocalteu reagent (10% in ultrapure water) was added and mixed. After 5 min of incubation, 120 µL of 20% NaCO_3_ was added, mixed and incubated during 1 h under constant agitation. Then, the respective absorbance values were measured at 760 nm in a multimode microplate reader (Synergy H1, Biotek Instruments, Winooski, VT, USA). Results were expressed as mg gallic acid equivalents per gram of extract (GAE/g extract), according to the respective calibration curve (10–600 µg/mL of gallic acid in 80% methanol).

#### 2.4.2. Total Flavonoid Content (TFC)

Total flavonoid content was measured following a previous methodology [24]. Briefly, 30 µL of each sample were mixed with 10 µL of 10% AlCl_3_, 10 µL of 1 M sodium acetate (C_2_H_3_NaO_2_), and 250 µL of ultrapure water in a microplate well and incubated at room temperature for 30 min. Absorbance was measured at 415 nm using a multimode microplate reader (Synergy H1, Biotek Instruments, Winooski, VT, USA). Results were expressed as quercetin equivalents per gram of extract (QE/g extract), based on a calibration curve derived from a quercetin solution (10–1000 µg/mL in 80% methanol).

#### 2.4.3. Antioxidant Activity

The antioxidant activity of the extracts was assessed using various assays for antioxidant capacity, as described in our previous work [24], with some modifications.

The scavenging activity against the 2,2-diphenyl-1-picrylhidrazyl (DPPH) free radical was performed using gallic acid as a reference standard for calibration (10–1000 µg/mL in 80% methanol). Briefly, a 50 µL aliquot of each extract (600 µg/mL of NaDES) was mixed with 150 µL of DPPH solution (0.1 mM in methanol). The mixture was stirred and incubated at 37 °C for 30 min. Absorbance was measured at 515 nm. Results were expressed as IC_50_ values (µg/mL) calculated by linear regression analysis.

The Ferric Reducing Antioxidant Power reagent (FRAP) reagent was prepared by mixing 300 mM acetate buffer (pH 3.6) and 10 mM solution of 2,4,6-tripiridil-s-triazine (TPTZ) in 40 mM HCl. A volume of 20 µL of each extract was mixed with 150 µL of FRAP reagent. After 15 min of incubation, absorbance was measured at 593 nm. The antioxidant/reducing capacity of the different extracts was expressed as µmol Trolox equivalents per gram of extract (calibration curve: 10–500 µg/mL of Trolox in methanol).

The oxygen radical absorbance capacity (ORAC) assay was used to assay the effect of the extracts on the decline in the fluorescence (λ_em_ = 528 nm; λ_ex_ = 485 nm) of fluorescein (1 µM) in the presence of the presence 2,2′-Azobis(2-methylpropionamidine) dihydrochloride radical (AAPH, 250 µM). Fluorescence was measured every 2 min for 2 h, and the result were expressed as µmol of Trolox equivalents (TE) per gram of extract, according to the respective calibration curve (10–100 µmol of Trolox in methanol).

### 2.5. Chromatographic Profile Using HPLC–DAD

#### 2.5.1. HPLC-DAD Analysis

Chromatographic analyses of the extracts were performed in a High-Performance Liquid Chromatography (HPLC) system (VWR-Hitachi Chromaster modular system, VWR International Ltd., Leicestershire, UK), consisting of a 5110 pump, 5430 diode array detector (DAD), 5310 column oven, 5260 autosampler and the Chromaster System Manager v2.30 software. The analyses were performed with a Symmetry C18 column, 5 µm particle size, 250 × 4.6 mm, (Waters Corporation, Milford, MA, USA) maintained at 30 °C, using a linear gradient solvent system consisting of ultrapure water, formic acid, and acetonitrile (87:5:3, *v*/*v*/*v*, solvent A) and ultrapure water, formic acid, and acetonitrile (40:5:50, *v*/*v*/*v*, solvent B) following a previously described method [25]. Initial conditions were 95% A and 5% B, then, the solvent ratio was changed to 75% A and 25% B in 50 min and returned to the initial conditions at min 55. The column was stabilized for an additional 10 min under the same gradient (95:5, A:B) before the next injection. The flow rate was set at 0.8 mL/min, and the injected volume was 10 μL. Samples were injected in duplicate.

Before injection, 50 to 90 mg of the extracts (supernatant) were weighed and diluted to a final volume of 1 mL using the mobile phase in initial conditions (95:5, A:B) to achieve a concentration of 50 to 90 mg/mL in the injected aliquot. The solutions were filtered using Polyvinylidene fluoride (PVDF) syringe filters (0.45 μm). Rutin, quercetin, gallic acid, and cyanidin-3-*O*-glucoside were used as standards at a concentration of 0.55 mg/mL, dissolved in the mobile phase mixture at the initial ratio.

UV-Vis spectra were recorded from 200 to 600 nm for peak characterization and the compounds were monitored at 280, 325, 354 and 520 nm. The retention time of each compound and its absorption spectrum were recorded and compared with standards and between samples to analyze differences in metabolite profiles according to the extraction solvent.

#### 2.5.2. Extraction Performance Analysis

To preliminarily assess the extraction performance of phenolic compounds, the areas under the curve (AUC, in arbitrary units) of selected chromatographic peaks were determined at their respective maximum absorbance wavelengths. Peaks, based on the UV-vis spectra, were grouped into three main families: phenolic acids, flavonoids, and anthocyanins [26].

In the first approach, the AUCs of individual peaks were summed for each compound family and extraction system, providing the total AUC per family for each solvent. This allowed an initial evaluation of the extraction efficiency of each solvent type for specific classes of compounds.

The second analysis considered the total AUC of all compound families within each extraction system as 100%, and the relative contribution of each family was calculated. This assessment provided insight into the distribution of compound families within each extraction system.

Finally, in a third analysis, representative compounds from each family were selected, and their AUCs were summed per extraction system, with the total set as 100%. This enabled comparisons of the relative abundance of individual compounds within and across extraction systems. The same procedure was applied to each family, allowing for both intra- and inter-family comparisons.

### 2.6. Multivariate Statistical Analyses

The statistical analysis was performed in the software IBM^®^ SPSS^®^ Statistics 22.0. All the experiments were repeated at least three times, except the chromatographic profiles. The results were expressed as the mean ± standard deviation (SD). One-way analysis of variance (ANOVA) and subsequently, Tukey’s multiple comparison post-hoc test (*p* < 0.05) was applied to determine statistically significant differences for spectrophotometric analyses among the different treatments. Additionally, to determine the spatial distribution and non-random patterns of the obtained extracts, a Principal Component Analysis (PCA) was performed based on the spectrophotometric and chromatographic analyses. To establish possible correlations between variables, a bivariate Pearson’s correlation analysis was carried out (95% confidence). The respective correlation coefficient (Pearson’s *r*) and significance value (*p*) were registered in each case.

## 3. Results and Discussion

### 3.1. Total Phenolics, Total Flavonoids, and Antioxidant Activity

Deep eutectic solvents have been considered to be green solvents due to their remarkable solubilizing power and have been recommended for different applications [20]. In this case, the NaDES-obtained extracts (M1–M7) appeared gelatinous and highly viscous, which is beneficial for direct analysis [27]. Conversely, the extracts made using acidified methanol (conventional extraction method, M8) were dried to eliminate the organic solvent before being subjected to the same analyses. According to Table 2, the phenolic and flavonoid content was variable among the different extraction strategies. The total phenolic content varied from 3.19 to 64.87 mg GAE/g extract, while the flavonoid content was between 0.94 to 35.38 mg QE/g extract. The samples exposing higher phenolic contents were the extracts obtained by using the extraction mixtures M2 (choline chloride:1,2-Propanediol, 1:2) followed by M6 (choline chloride:lactic acid, 1:2), with 64.87 ± 4.85 and 19.58 ± 1.38 mg GAE/g extract, respectively. The same tendency was observed for the total flavonoid content, with the extraction mixtures M2 and M6 exposing the highest values (35.38 and 10.64 mg QE/g of dry extract). In line with the present results, it has been reported that choline chloride in combination with 1,3-propanediol and lactic acid showed the highest efficiency in the extraction of phenolic compounds in plant sources such as manuka leaves [28].

On the other hand, some of the lowest phenolics and flavonoid contents were revealed by the conventional extraction using acidified methanol (M8), with 4.10 mg GAE/g extract and 1.37 mg QE/g extract, respectively. These values were statistically comparable (*p* < 0.05) to the extraction mixtures M1 and M5, obtained by extracting with choline chloride: glycerol (1:2) and choline chloride: oxalic acid (1:1), respectively, which showed the lowest phenolic and flavonoid content. For comparison with other studies employing conventional extraction methods, the ethanolic extracts of *Ugni molinae* leaves exhibited phenolic and flavonoid content between 158 to 260 mg GAE/g dry extract and from 30 to 53 mg QE/g dry extract [29]. While the study indicates a higher phenolic content, it is important to note that the flavonoid levels are on par with the elevated values found using the NaDES extraction mixture M2, as previously stated. However, directly comparing these results is challenging due to variations in the presentation of their findings and the different parts of the plant they utilized. This underscores the need for careful consideration when interpreting these results.

In the same way, the antioxidant activity of the obtained extracts was also different among the extraction strategies in NaDES. As shown in Table 2, the highest global antioxidant activity was observed by using the extraction mixture M2, with an IC_50_ of 1.05 µg extract/mL through the DPPH free radical scavenging method, and 40,291 µmol TE/g extract for the ORAC assay. These results are concordant with the higher phenolic and flavonoid content for the same extract. However, the conventional extraction using methanol: formic acid (95:5), accounted for the highest antioxidant activity in terms of the FRAP and ORAC assays, with 45.91 and 568,851 µmol TE/g extract, respectively, along with a low IC_50_ measured by the DPPH assay in that sample (3.62 µg/mL). Nevertheless, ethanolic extracts of *Ugni molinae* fruits have been reported to exhibit an antioxidant activity of approximately 3300 µmol Trolox/g extract in the ORAC assay, which is about ten times lower than the values obtained using NaDES extraction (Table 2) [30]. Although a significant correlation between the phenolic content and the antioxidant capacity has been reported for plant extracts [31], in this case, all the extracts showed a high antioxidant activity, but the results are not necessarily concordant between the different techniques. The type of phenolic compounds included in the extracts, the corresponding limitations or interferences of each technique, or even the sample sources could all be responsible for these results [31,32]. However, the present results reflect a significant phenolic content and antioxidant activity, which is concordant with other reports, even though using different in vitro methodologies [33]. This indicates that while the results are optimistic, more research is needed to confirm the extracts’ biological activity. According to previous works, the antimicrobial activity of murta fruits [30] would also be a valuable issue to assess in further research.

Beyond their antioxidant capacity, the flavonoid- and anthocyanin-type compounds detected in *U. molinae* fruits are widely recognized for their positive biological effects. Flavonol derivatives such as those tentatively assigned to quercetin, kaempferol, and myricetin have been reported to exert neuroprotective actions by reducing protein aggregation in models of neurodegenerative diseases [34], while anthocyanin-type compounds contribute not only to antioxidant defense but also to the modulation of inflammatory processes [8]. Previous studies have also shown that phenolic extracts from *U. molinae* may inhibit key enzymes of carbohydrate metabolism, suggesting potential benefits in hyperglycemia management [35], in addition to antimicrobial and tyrosinase-inhibiting activities [10]. Altogether, this evidence underscores that the compound families enriched in our extracts hold practical relevance for nutraceutical, pharmaceutical, and cosmetic applications

### 3.2. HPLC-DAD

**Figure 1:**
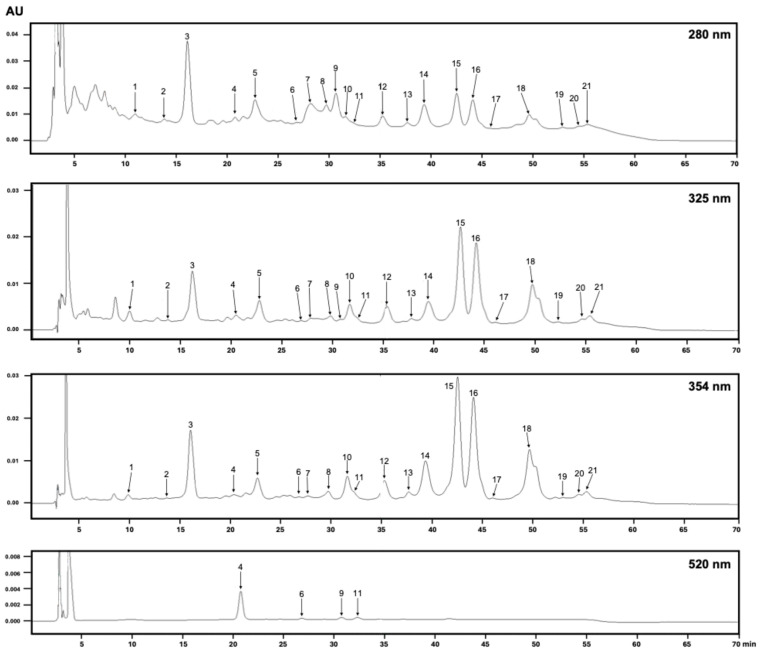
HPLC-DAD chromatograms of the extract M8 obtained from the fruits of *U. molinae*. The extract was analyzed at four wavelengths (280, 325, 354, and 520 nm). The numbers of each peak correspond to those in Table 3.

The HPLC-DAD compound profiles of the M8 extract and the extracts obtained using NaDES were compared. Figure 1 displays the chromatograms obtained from M8 at the four analyzed wavelengths. Table 3 summarizes the retention times and UV-vis absorption spectra of each compound and compares their presence in each extract. The numbering used to identify each compound in Figure 1 corresponds to the same numbers in Table 3.

**Figure 2:**
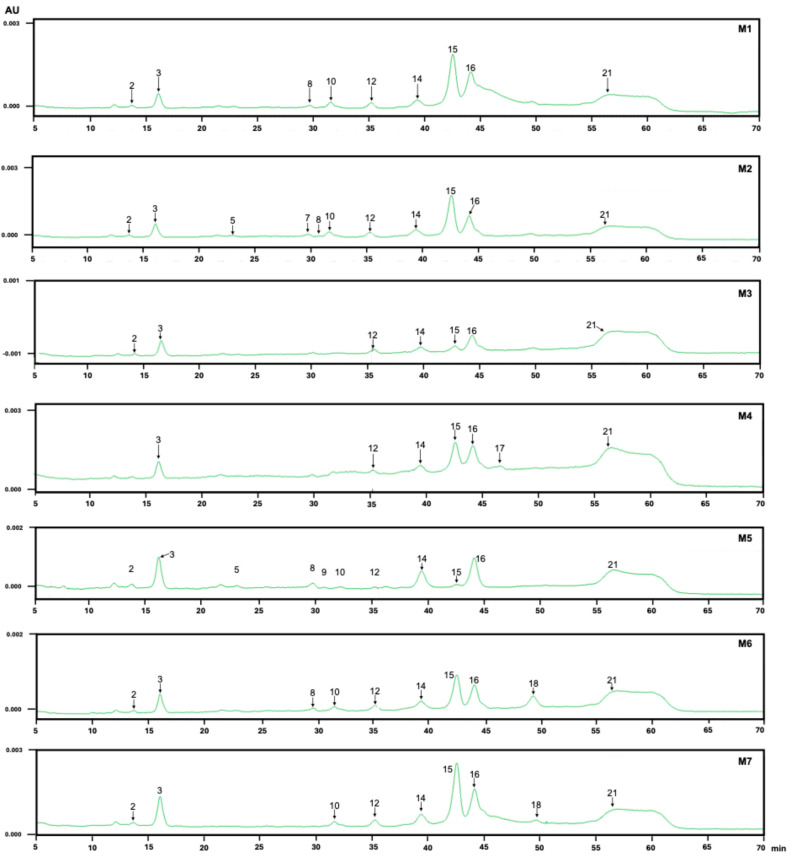
HPLC-DAD chromatograms of the extracts obtained at 325 nm from *U. molinae* fruits using NaDESs as extraction solvents. M1–M7 solvent mixture compositions are described in Table 1. The numbers for each peak correspond to those in Table 3. Each extract was analyzed at four wavelengths (280, 325, 354, and 520 nm) and the chromatograms are presented in Figure S1.

The spectral data obtained from the DAD enabled the grouping of the detected compounds into families that share similar absorption maxima and spectral shapes, thereby facilitating the comparison of the chromatographic profile among the different extracts. This study examined the UV-Vis spectra of 21 chromatographic peaks to ensure that each one corresponded to a single compound rather than an overlap of multiple peaks, except for peak 21, which was considered broader to determine the peak determination (Figure 2). This approach provided a more precise compound profile than other preliminary chromatographic techniques, such as thin-layer chromatography.

The methanol: formic acid mixture extracts more compounds than the NaDES extracts, as observed in the number of chromatographic peaks at the different wavelengths analyzed (Figure 1, Table 3).

At a wavelength of 520 nm, four compounds (**4**, **6**, **9** and **11**) were observed, which are assumed to be anthocyanin derivatives, considering the UV spectra of each compound. Only M-8 extract contained all four anthocyanin-like compounds (Table 3, Figure 1). Compared to NaDES extracts, only compounds **4** and **9** were present, except for M-4, which included only **9** (Table 3, Figure 2). Although NaDES mixtures can extract anthocyanins, they are extracted in smaller numbers (2 or 1 out of 4 compounds extracted with the methanol: formic acid) and in smaller quantities than the acidified methanol. This can be seen by the lower height of the peaks measured in absorbance units (AU). The amounts should be confirmed by quantification using a calibration curve obtained with the area under the integrated curve of the peaks. Although cyanidin-3-glucose was used as a reference compound to compare retention time and UV-Vis absorption spectra, it was inconclusive for assigning identity.

It has been observed that the antioxidant activity of M-8 and NaDES extracts could be attributed to other compounds rather than anthocyanins. This is consistent with findings from other native fruits of the genus Gaultheria [36,37], where co-pigments or flavonoids contributed more to the antioxidant capacity of the extracts. In the case of extracts derived from fruits of eight genotypes of *U. molinae*, quercetin derivatives such as isorhamnetin, kaempferol, and myricetin were identified [34]. This was confirmed by Ordoñez et al. (2022), who utilized HPLC-MS/MS to analyze *U. molinae* fruit extracts [35].

Compounds **1** and **2** have λ max of 276 and 278 nm, respectively. Their retention time and absorption spectra suggest that these are phenolic compounds of the hydroxybenzoic acid type, such as gallic acid or glucoside derivatives [29]. Compound **3** is present in all extracts, in varying amounts, and could be considered a potential marker for future comparisons. Compounds **3**, **5**, **7**, **8**, **10** and **12**–**21** have λ max values between 352 and 363, indicating a more significant presence of flavonoid-type compounds. As per previous literature reports, these may be of the quercetin, kaempferol, or myricetin core [38]. Pérez-Arancibia et al. (2021) reported that the compounds found in extracts from murta fruits were glycoside derivatives of quercetin and myricetin [34]. Additionally, glycoside derivatives of other flavonoids, such as kaempferol and isorhamnetin, ellagitannins like HHDP-galloyl hexose, and phenolic acids, such as caffeic acid derivatives, were identified.

The increase in the baseline of the M-8 chromatogram during the first 10 min is more pronounced when observed at 280 nm. This observation may be attributed to the presence of a wider variety of proanthocyanidin-like compounds, catechin oligomers, and epicatechin oligomers, which are commonly found in red fruits and may contribute to antioxidant capacity [25]. These compounds are also known to produce this baseline phenomenon. Moreover, the absence of this effect at other wavelengths supports the decision to analyze each extract at four different wavelengths.

The significant rise in the baseline towards the end of the chromatograms from M-1 to M-7, particularly evident in the broadening of peak **21** (Figure 2), may result from the overlap of compounds with similar polarities, which the current method struggles to resolve. Although there is potential for improving this limitation, analyzing all samples using a single methodology was necessary for comparison. This pattern was consistently observed in all samples extracted with NaDES, as the overlapping compounds were highly likely to belong to the same structural type.

HPLC is the leading technique for analyzing polyphenols in fruits. Its capability for studying complex mixtures makes it an essential tool for characterizing phenolic profiles and generating distinctive “fingerprints” within food matrices [39]. The choice of the detector to be coupled to the liquid chromatography system depends on the specific analytical goals. Fluorescence detectors are well-suited for sensitive detection of specific phenolic compounds, while diode array UV-Vis (DAD) detectors provide broader spectral information for compound identification [40]. Mass spectrometry (MS) detectors can be used for in-depth structural characterization, providing molecular fingerprints of individual analytes [41]. This variety of detection options allows researchers to tailor their LC analyses to specific needs.

HPLC coupled to a diode array detector is a useful analytical tool to characterize polyphenols. This combination provides complementary information on the polarity and structural features of these diverse compounds. Retention time is a measure of the affinity of a molecule for the stationary phase, providing an indirect measure of its polarity [42]. The UV-Vis absorption spectra, captured by the DAD, reflect the electronic transitions within the aromatic rings of phenolic compounds. These spectral fingerprints can be used to distinguish between different classes and sub-groups of phenolics. For example, anthocyanins absorb at 500–550 nm, while flavonols and flavones absorb between 300 and 380 nm [41].

A future identification by HPLC-DAD-MS/MS will allow for confirmation of the identity of the compounds. Therefore, it will be possible to quantify the main compounds and to apply statistical analyses that will enable the search for existing correlations between the presence or absence of a compound and the antioxidant capacity of an extract. It will also allow the establishment of quantitative differences between the extracts associated with a specific compound since it has been possible to visually demonstrate the similarities and differences in the profile of secondary metabolites between extracts.

### 3.3. Extraction Performance Analysis

**Figure 3:**
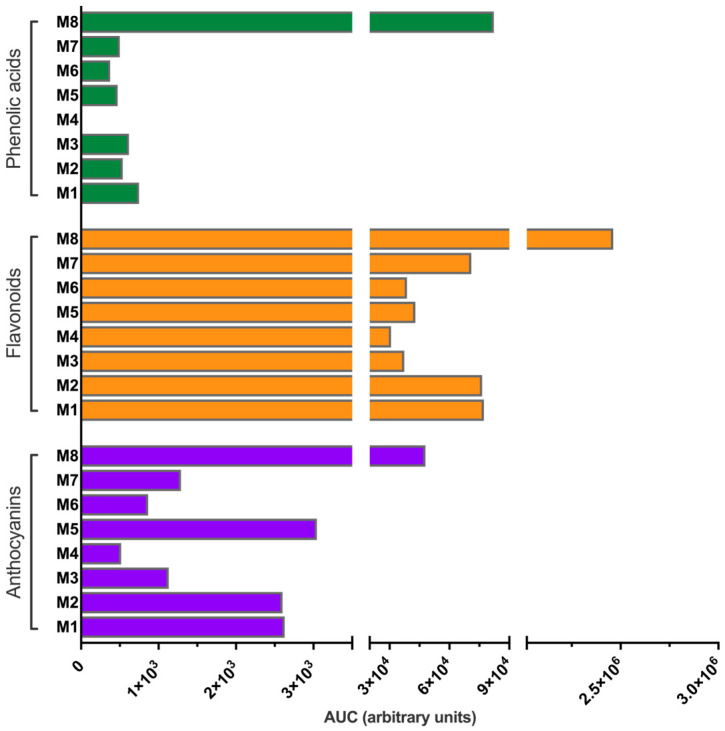
Sum of the AUCs of individual peaks for each compound family and extraction system. M1-M8 solvent mixture compositions are described in Table 1. The *Y*-axis was divided into three non-continuous sections to allow for a clear visualization of the differences between the data, including those of smaller magnitude. The AUCs of each of the compounds classified by the type of solvent mixture are shown in Table S1.

The extraction efficiency of each compound family using different solvent systems was preliminarily evaluated based on the AUC obtained from each chromatogram (Figure 3). The results indicate that, across all compound types, M8 was the most effective extraction solvent, followed by M1, M2, and M7 for phenolic acids and flavonoids, and by M5, M1, and M2 for anthocyanins. These findings are consistent with the observations reported by Fuentes-Jorquera et al. (2024), who also identified higher yields using conventional solvents compared to NaDES systems [26].

Table 4 presents the distribution of extracted compounds by family. To determine the percentages, we first calculated the total AUC for each family (phenolic acids, flavonoids, and anthocyanins) separately for each extraction system. The total AUC for each system was then considered as 100%. Subsequently, we calculated the percentage of each family relative to this 100% for each extraction system. A pronounced tendency towards flavonoid extraction was observed in both NaDES and conventional extraction systems. In the analysis of relative composition within each extraction system, M1, M2, M3, and particularly M5 exhibited higher extraction yields of anthocyanins compared to the conventional solvent mixture (M8), with M5 achieving approximately 5% more. In contrast, the extraction of phenolic acids was consistently lower across all NaDES systems compared to the conventional solvent M8.

**Figure 4:**
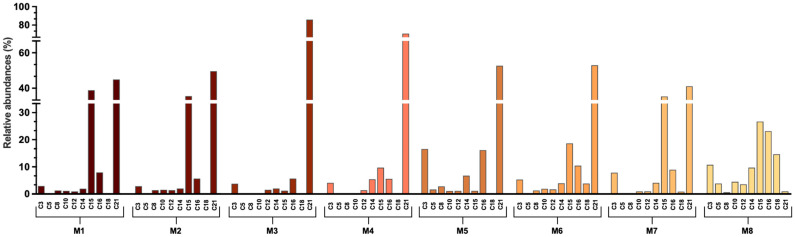
Relative abundance of the representative compounds from the extracted flavonoids. M1–M8 solvent mixture compositions are described in Table 1. The retention times of each compound and its corresponding absorption spectra are shown in Table 3. The *Y*-axis was divided into three non-continuous sections to allow for a clear visualization of the differences between the data, including those of smaller magnitude. Percentage values for each compound are shown in Table S3.

**Figure 5:**
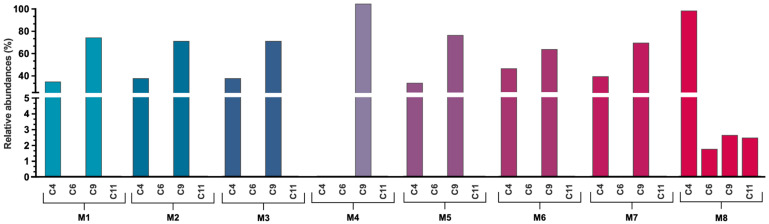
Relative abundance of the representative compounds from the extracted anthocyanins. M1–M8 solvent mixture compositions are described in Table 1. The retention times of each compound and its corresponding absorption spectra are shown in Table 3. The *Y*-axis was divided into two non-continuous sections to allow for a clear visualization of the differences between the data, including those of smaller magnitude. Percentage values for each compound are shown in Table S4.

Figure 4 and Figure 5 illustrate the relative abundance of flavonoid-type compounds and anthocyanins, respectively, for each extraction system. Data on phenolic acids are presented in Table S5.

For flavonoids, compounds **7**, **13**, **17**, **19**, and **20** were excluded from the analysis due to their low representation, comprising less than 5% of the total extracted flavonoids. In M8, these compounds accounted for 1.58% and in M4, 3.20%. The remaining compounds represented over 95% of the extracted flavonoids (Figure 4, Table S3).

A broader distribution of flavonoid compounds was observed when M8 was employed as the extraction solvent. In contrast, NaDES systems exhibited a preferential extraction of more apolar compounds, as inferred from the HPLC gradient and retention times, predominantly represented by compound **21**. In most NaDES systems, compound **21** constituted over 45% of the extracted flavonoids, reaching 70% in M4 and 85% in M3. Excluding **21**, NADESs primarily extracted compounds **15** and **16** in varying proportions. Specifically, in M1, M2, and M7, these compounds accounted for approximately 45% of the extracted flavonoids. In other NaDES systems, **14**, **15**, and **16** collectively represented 25–30% of the extracted flavonoids (Figure 4, Table S3).

For anthocyanins, as shown in Figure 5, only M8 enabled the extraction of four compounds within this family (**4**, **6**, **9**, and **11**), with **4** representing 93% of the total (Figure 5, Table S4). In NaDES systems, the extraction was limited to **4** and **9**, except in M4, where **9** constituted 100% of the extracted anthocyanins. Across the NaDES systems, a similar distribution pattern was observed, with **4** accounting for 30–40% and **9** for 60–70% of the extracted anthocyanins (Figure 5, Table S4).

In this study, compounds were grouped into families based on their UV-Vis absorption spectra. Although there was no tentative identification by mass spectrometry (MS) or complementary detectors such as fluorescence, this classification enabled a preliminary assessment of the types of compounds extracted by each system.

Unlike the findings of Fuentes-Jorquera et al. (2024) [26], where flavonoids were less extracted from *Ugni candollei* (white murta) fruits compared to hydroxycinnamic and hydroxybenzoic acids (phenolic acids), the present work showed a higher abundance of flavonoids. This may be attributed to differences in species or extraction conditions. Here, a broader diversity of NaDES mixtures was employed, alongside ultrasonic-assisted extraction and sample homogenization using an ultraturrax, which may have enhanced flavonoid release. Similar improvements have been observed in ultrasound-assisted NaDES extraction [43]. Moreover, Chemat et al. (2012) reported that ultrasound promotes cell wall disruption and solute diffusion, increasing extraction yields [44].

The M5 system appeared to be the most effective NADES for anthocyanins, particularly for compounds **4** and **9**, showing higher extraction than other NaDES systems (Figure 3, Table 4). This pattern aligns with the findings of Jovanović et al. (2022), who reported that specific NaDES systems favored the selective extraction of anthocyanins from Bilberry fruits (*Vaccinium myrtillus* L.), offering an innovative, sustainable, and eco-friendly method that could be an effective alternative to conventional anthocyanin extraction techniques [45].

Extraction of polyphenols and plant compounds in general is influenced by several factors, including solute-solvent interactions, matrix composition, and whether the compounds are present in free or bound forms [46]. Conventional solvents remain a benchmark when evaluating the efficiency of green extraction technologies such as NaDES. Differences between NaDES and conventional systems should not be interpreted solely in terms of superiority but rather in terms of selectivity. This selective extraction may enrich specific compound classes, potentially simplifying downstream isolation by reducing the need for additional purification steps [44,46].

### 3.4. Multivariate Statistical Analyses

**Figure 6:**
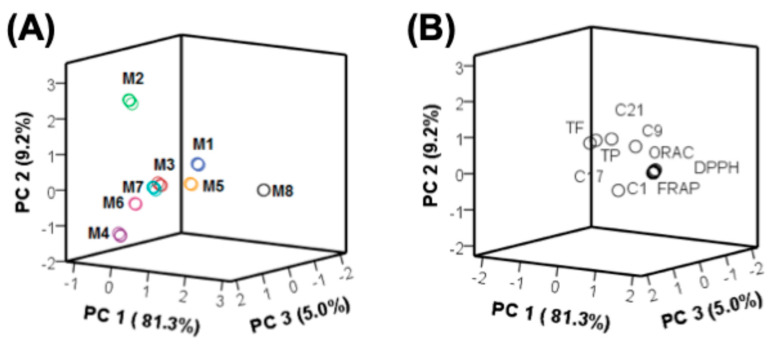
Principal Component Analysis (PCA). (**A**): score plot; (**B**): loading plot. M1–M8 solvent mixture compositions are described in Table 1.

A principal component analysis (PCA) was carried out to assess the spatial distribution of the extraction systems (M1–M8) and to explore the relationships among the TPC, TFC, antioxidant capacity (DPPH, FRAP, ORAC), and the relative abundance of the found signals in the chromatographic analysis. Three principal components (PCs) were extracted, explaining 95.5% of the total variability. As shown in Figure 6, the score plot shows a clear separation of samples according to the extraction mixtures. The extract M8 was separated along with PC1, indicating a distinct chemical profile compared to the NaDES-based systems. M1 and M5 clustered together, slightly separated from M8 and the other NaDES extracts, while M2, M3, M4, M6, and M7 formed a more compact cluster, suggesting compositional similarities among these systems (Figure 6A).

The distribution of variables in the loading plot (Figure 6B) indicates that the separation along PC1 was mainly influenced by antioxidant parameters (DPPH, FRAP, ORAC) and total phenolic content (TPC), all of which were closely associated with the M8 extract. Conversely, the cluster formed by M2–M7 was more strongly related to specific flavonoid-type compounds, suggesting that these were better extracted by selected NaDES systems.

The intermediate positioning of M1 and M5 along PC1 and PC2 (Figure 6A) suggests a broader extraction profile for these systems, likely reflecting a more balanced interaction between the solvent system and different compound classes. Notably, M5 showed a favorable extraction performance for anthocyanins such as **4** and **9**, while still allowing a substantial recovery of total phenolics and antioxidant capacity.

These results confirm that NaDES mixtures are not only viable alternatives to conventional extraction solvents but can also act as selective media for the enrichment of specific compound classes. The preferential extraction of flavonoids by NaDES, in contrast to the more polar phenolic compounds, highlights their potential application in targeted extraction strategies and downstream isolation protocols, as mentioned before. To support this observation, Pearson correlation coefficients (Pearson’s *r*) were calculated (Tables S1 and S2).

As shown in Table S6, strong correlations were observed between antioxidant capacity assays (FRAP, ORAC, and DPPH) and the major flavonoids **3**, **15**, and **16**, confirming their predominant contribution to the antioxidant profile of the different *murta* extracts (*r* > 0.87, *p* < 0.01). In addition, these compounds exhibited strong correlations among themselves and with other signals in the chromatographic profiles, suggesting consistent distribution patterns across the extracts. This highlights that the most abundant flavonoids are not only quantitatively relevant but also functionally decisive for the antioxidant properties of the extracts.

On the other hand, in Table S7, most of the compounds showed very strong correlations (*r* > 0.98, *p* < 0.01), indicating that their abundance patterns were highly consistent across the extracts. Among them, the anthocyanin **4** displayed significant positive correlations with the antioxidant capacity assays, suggesting a complementary role in the antioxidant response. Although other anthocyanins such as **9** did not show significant associations, their distribution followed similar extraction trends.

## 4. Conclusions

This work demonstrates that choline chloride–based NaDES represent an effective and sustainable alternative for extracting polyphenols from *Ugni molinae* fruits. Compared to conventional solvents, NaDES provided higher recovery of flavonoid-rich fractions and enhanced antioxidant activity, confirming their role as selective media for less polar bioactive compounds. The integration of UAE further improved performance, highlighting the value of combining green solvents with innovative extraction technologies. Chromatographic profiling revealed that NaDES preferentially enriched quercetin derivatives and related flavonoids, while certain formulations also favored the recovery of anthocyanins. Overall, these findings support NaDES as a promising tool for developing targeted extraction strategies aimed at obtaining high-value phytochemicals from Chilean fruits of economic importance. Future research should focus on assessing the stability, bioaccessibility, and biological efficacy of NaDES extracts, while also addressing limitations such as the reduced recovery of highly polar compounds like anthocyanins, to strengthen their potential applications in functional foods, nutraceuticals, and pharmaceutical formulations.

## Figures and Tables

**Table 1:** Extraction mixtures of Natural Deep Eutectic Solvents (NaDES) used in treatments M1 to M7, and acidified methanol (M8, control) employed in the extraction process.

Mixtures	Hydrogen Bond Acceptor	Hydrogen Bond Donor	Molar Ratio
M1	Choline chloride	Glycerol	1:2
M2	Choline chloride	1,2-Propanediol	1:2
M3	Choline chloride	D-glucose	1:2
M4	Choline chloride	Citric acid	1:2
M5	Choline chloride	Oxalic acid	1:1
M6	Choline chloride	Lactic acid	1:2
M7	Choline chloride	Urea	1:2
M8 (control)	Methanol: Formic Acid		95:5

**Table 2:** Total phenolic content (TPC), total flavonoid content (TFC), and antioxidant activity of *U. molinae* fruit extracts.

Treatment	TPC (mg GAE/g ext)	TFC (mg QE/g ext)	DPPH (IC_50_) (µg ext/mL)	FRAP (µmol TE/g ext)	ORAC (µmol TE/g ext)
M1	3.19 ± 0.12 ^a^	3.16 ± 0.76 ^b^	2.15 ± 0.04 ^g^	2.45 ± 0.11 ^a^	39,073 ± 3 ^e^
M2	64.87 ± 4.85 ^c^	35.38 ± 0.76 ^e^	1.05 ± 0.03 ^d^	3.12 ± 0.15 ^a^	40,291 ± 4 ^g^
M3	7.43 ± 1.73 ^a^	7.81 ± 0.76 ^c^	1.42 ± 0.04 ^f^	2.39 ± 0.05 ^a^	38,428 ± 6 ^b^
M4	6.32 ± 3.68 ^a^	8.52 ± 1.38 ^cd^	1.20 ± 0.02 ^e^	3.05 ± 0.06 ^a^	38,356 ± 3 ^a^
M5	8.00 ± 0.5 ^a^	0.94 ± 0.61 ^a^	0.94 ± 0.03 ^c^	3.50 ± 0.15 ^a^	38,948 ± 9 ^d^
M6	19.58 ± 1.38 ^b^	10.64 ± 0.30 ^d^	0.78 ± 0.04 ^b^	3.52 ± 0.11 ^a^	39,168 ± 3 ^f^
M7	8.78 ± 2.67 ^a^	8.92 ± 0.76 ^cd^	0.68 ± 0.01 ^a^	4.91 ± 0.21 ^a^	38,920 ± 6 ^c^
M8	4.10 ± 0.36 ^a^	1.37 ± 0.03 ^ab^	3.62 ± 0.02 ^h^	45.91 ± 6.87 ^b^	568,851 ± 4 ^h^

M1–M8 solvent mixture compositions are described in Table 1. DPPH: 2,2-diphenyl-1-picrylhydrazyl radical; FRAP: ferric reducing antioxidant power; ORAC: oxygen radical antioxidant capacity; ext: extract; GAE: gallic acid equivalents; QE: quercetin equivalents; IC_50_: extract concentration scavenging 50% of the DPPH radical. TE: Trolox equivalents. Results are the mean values ± SD of three independent experiments. Different superscript letters (a–h) in the same column show significant differences within each assay according to Tukey’s test (*p* < 0.05).

**Table 3:** Retention time and UV spectra of the main compounds in the *Ugni molinae* extracts.

Peak	UV-Vis Spectra	λmax (nm)	λused (nm)	t_R_ (min)	M8	M1	M2	M3	M4	M5	M6	M7
1	244, 276	276	280	10.967	x	-	-	-	-	-	-	-
2	244, 278	278	280	13.833	x	x	x	x	-	x	x	x
3	244, 276, 352	352	354	16.087	x	x	x	x	x	x	x	x
4	244, 276, 364, 513	513	520	20.773	x	x	x	x	-	x	x	x
5	244, 278, 352, 370 sh	352	354	22.720	x	-	-	-	-	x	-	-
6	244, 280, 359, 518	518	520	26.807	x	-	-	-	-	-	-	-
7	244, 276, 356	356	354	27.713	x	-	-	-	-	-	-	-
8	244, 276, 356	356	354	29.720	x	-	-	-	-	x	x	-
9	244, 266, 300, 520	520	520	30.767	x	x	x	x	x	x	x	x
10	244, 272, 354	354	354	31.620	x	x	x	-	x	x	x	x
11	244, 352, 520	520	520	32.313	x	-	-	-	-	-	-	-
12	244, 272, 354	354	354	35.267	x	x	x	x	x	x	x	x
13	244, 272, 354	354	354	37.687	x	-	-	-	-	-	-	-
14	245, 304, 363	363	354	39.367	x	x	x	x	x	x	x	x
15	244, 268 sh, 352	352	354	42.527	x	x	x	x	x	x	x	x
16	244, 270 sh, 351	351	354	44.107	x	x	x	x	x	x	x	x
17	244, 270 sh, 351	351	354	45.953	x	-	-	-	x	-	-	
18	244, 270 sh, 352	352	354	49.653	x	-	-	-	-	-	x	x
19	244, 270 sh, 352	352	354	52.940	x	-	-	-	-	-	-	-
20	244, 270 sh, 352	352	354	54.527	x	-	-	-	-	-	-	-
21	244, 270 sh, 352	352	354	55.353	x	x	x	x	x	x	x	x

M1–M8 solvent mixture compositions are described in Table 1; λmax: wavelength (in nm) of maximum absorption; λused: wavelength (in nm) used to obtain the UV-vis spectra; t_R_: retention time in minutes; x: peak present in the sample; -: peak not found in the sample; nm: nanometers; sh: shoulder.

**Table 4:** Relative contribution, in percentages, of the different polyphenol families within each extraction system.

Family	M1	M2	M3	M4	M5	M6	M7	M8
Phenolic acids	0.93	0.68	1.58	0.00	1.02	0.93	0.69	3.17
Flavonoids	95.81	96.06	95.54	98.35	92.42	96.90	97.54	94.98
Anthocyanins	3.26	3.27	2.89	1.65	6.56	2.17	1.77	1.85

M1–M8 solvent mixture compositions are described in Table 1. Values shown in the table are in percentages. The sum of each column is 100%.

## Data Availability

The original contributions presented in this study are included in the article/Supplementary material. Further inquiries can be directed to the corresponding authors.

## Supplementary Materials

The following supporting information can be downloaded at: https://www.mdpi.com/article/10.3390/antiox14101234/s1; Table S1: AUC of each of the compounds classified by the type of solvent mixture used for extraction; Table S2: Total AUC per compound family; Table S3: Relative abundance, in percentages, of the representative compounds from the extracted flavonoids; Table S4: Relative abundance, in percentages, of the representative compounds from the extracted anthocyanins; Table S5: Relative abundance, in percentages, of the representative compounds from the extracted phenolic acids; Table S6: Correlation matrix (Pearson’s *r* coefficients) between variables obtained from spectrophotometric analysis (TPC, TFC, FRAP, DPPH, ORAC) and chromatographic profiles (flavonoid related compounds). Significant correlations are indicated with * (*p* < 0.05) and ** (*p* < 0.01); Table S7: Correlation matrix (Pearson’s *r* coefficients) between variables obtained from spectrophotometric analysis (TPC, TFC, FRAP, DPPH, ORAC) and chromatographic profiles (phenolic acids and anthocyanins related compounds). Significant correlations are indicated with * (*p* < 0.05) and ** (*p* < 0.01); Figure S1: *Ugni molinae* fruits. The maps shows the place of collection of the ripe fruits. Figure S2. HPLC-DAD chromatograms of the extracts obtained at 280, 325, 354, and 520 nm from *U. molinae* fruits using NaDES as extraction solvents. M1-M7 solvent mixture composition are described in Table 1. The numbers of each peak are the same as Table 3.
