# Supplementary material for: Natural Deep Eutectic Solvent (NaDES) Extraction, HPLC-DAD Analysis, and Antioxidant Activity of Chilean Ugni molinae Turcz. Fruits
Source: Antioxidants (Basel). 2025 Oct 14;14(10):1234. doi: 10.3390/antiox14101234 (PMC12561733; doi:10.3390/antiox14101234)
Supplement: Supplementary file 1 [file antioxidants-14-01234-s001.zip › antioxidants-3885717-supplementary.pdf]

Supplementary Materials

Article

Natural Deep Eutectic Solvent (NaDES) Extraction, HPLC-DAD Analysis, and Antioxidant Activity of Chilean *Ugni molinae* Turcz. Fruits

Javier Antileo-Laurie [1], Verónica Olate-Olave [2], Valentina Fehrmann-Riquelme [3], Camila Anabalón-Alvarez [3], Luis Cid-Carrillo [3], Javier Campanini-Salinas [4], Carlos Fernández-Galleguillos [5,*] and Luisa Quesada-Romero [3,*]

[1] Escuela de Química y Farmacia, Facultad de Ciencias, Universidad San Sebastián, General Lagos 1163, Valdivia 5090000, Chile; jantileol@correo.uss.cl
[2] Departamento de Bioquímica Clínica e Inmunohematología, Universidad de Talca, Avenida Lircay s/n, Talca 3460000, Chile; volate@utalca.cl
[3] School of Nutrition and Dietetics, Faculty of Rehabilitation and Quality of Life Sciences, Universidad San Sebastían, General Lagos 1163, Valdivia 5090000, Chile; vfehrmannr@correo.uss.cl; canabalona1@correo.uss.cl; lcidc1@correo.uss.cl; luisa.quesada@uss.cl (L.Q-R.)
[4] Escuela de Química y Farmacia, Facultad de Ciencias, Universidad San Sebastián, Lago Panguipulli 1390, Puerto Montt 5501842, Chile; javier.campanini@uss.cl
[5] Departamento Biomédico, Facultad de Ciencias de la Salud, Universidad de Antofagasta, Antofagasta 1270300, Chile; carlos.fernandez@uantof.cl (C. F-G)
* Correspondence: luisa.quesada@uss.cl (L.Q-R.); carlos.fernandez@uantof.cl (C.F-G.)

Table S1. AUC of each of the compounds classified by the type of solvent mixture used for extraction.

Sample ID	C1	C2	C3	C4	C5	C6	C7	C8	C9	C10	C11	C12	C13	C14	C15	C16	C17	C18	C19	C20	C21
M1	0	749	2,252	795	0	0	0	986	1,834	889	0	713	0	1,503	30,038	6,143	0	0	0	0	34,761
M2	0	537	2,160	867	0	0	0	1,071	1,733	1,167	0	1,075	0	1,525	27,158	4,323	0	0	0	0	37,906
M3	0	617	1,401	377	0	0	0	0	754	0	0	567	0	752	442	2,114	0	0	0	0	32,126
M4	0	0	1,262	0	0	0	0	0	518	0	0	417	0	1,669	2,992	1,717	985	0	0	0	21,749
M5	0	472	7,114	872	697	0	0	1,193	2,174	476	0	498	0	2,907	484	6,936	0	0	0	0	22,610
M6	0	374	2,062	359	0	0	0	490	508	733	0	648	0	1,521	7,225	4,053	0	1,496	0	0	20,548
M7	0	500	5,556	451	0	0	0	0	840	670	0	702	0	2,896	25,086	6,316	0	587	0	0	29,164
M8	55,471	26,752	265,046	44,681	94,626	841	1,842	15,994	1,254	110,446	1,177	86,892	24,018	239,114	657,398	570,014	571	360,545	2,596	9,876	23,634

The AUC are expressed in arbitrary units. The M1-M8 solvent mixture compositions are described in Table 1.

Table S2. Total AUC per compound family.

Family	Total AUC	
**PHENOLIC ACIDS** (corresponds to C1 added to C2)	M1	749.00
	M2	537.00
	M3	617.00
	M4	-
	M5	472.00
	M6	374.00
	M7	500.00
	M8	82,223.00
**FLAVONOIDS** (is the sum of C3, C5, C7, C8, C10, C12 to C21)	M1	77,285.00
	M2	76,385.00
	M3	37,402.00
	M4	30,791.00
	M5	42,915.00
	M6	38,776.00
	M7	70,977.00
	M8	2,462,612.00
**ANTHOCYANINS** (is the sum of C4, C6, C9, C11)	M1	2,629.00
	M2	2,600.00
	M3	1,131.00
	M4	518.00
	M5	3,046.00
	M6	867.00
	M7	1,291.00
	M8	47,953.00

AUC in arbitrary units. M1-M8 solvent mixture composition are described in Table 1. The data shown in this table is associated with Figure 3.

Table S3. Relative abundance, in percentages, of the representative compounds from the extracted flavonoids.

Sample ID	C3	C5	C7	C8	C10	C12	C13	C14	C15	C16	C17	C18	C19	C20	C21	Total flavonoids
M1	2.91	0.00	0.00	1.28	1.15	0.92	0.00	1.94	38.87	7.95	0.00	0.00	0.00	0.00	44.98	100
M2	2.83	0.00	0.00	1.40	1.53	1.41	0.00	2.00	35.55	5.66	0.00	0.00	0.00	0.00	49.62	100
M3	3.75	0.00	0.00	0.00	0.00	1.52	0.00	2.01	1.18	5.65	0.00	0.00	0.00	0.00	85.89	100
M4	4.10	0.00	0.00	0.00	0.00	1.35	0.00	5.42	9.72	5.58	3.20	0.00	0.00	0.00	70.63	100
M5	16.58	1.62	0.00	2.78	1.11	1.16	0.00	6.77	1.13	16.16	0.00	0.00	0.00	0.00	52.69	100
M6	5.32	0.00	0.00	1.26	1.89	1.67	0.00	3.92	18.63	10.45	0.00	3.86	0.00	0.00	52.99	100
M7	7.83	0.00	0.00	0.00	0.94	0.99	0.00	4.08	35.34	8.90	0.00	0.83	0.00	0.00	41.09	100
M8	10.76	3.84	0.07	0.65	4.48	3.53	0.98	9.71	26.70	23.15	0.02	14.64	0.11	0.40	0.96	100

M1-M8 solvent mixture compositions are described in Table 1. The retention times of each compound and its corresponding absorption spectra are shown in Table 3. The data shown in this table is associated with Figure 4.

Table S4. Relative abundance, in percentages, of the representative compounds from the extracted flavonoids.

Sample ID	C4	C6	C9	C11	Total anthocyanins
M1	30.24	0.00	69.76	0.00	100
M2	33.35	0.00	66.65	0.00	100
M3	33.33	0.00	66.67	0.00	100
M4	0.00	0.00	100.00	0.00	100
M5	28.63	0.00	71.37	0.00	100
M6	41.41	0.00	58.59	0.00	100
M7	34.93	0.00	65.07	0.00	100
M8	93.18	1.75	2.62	2.45	100

M1-M8 solvent mixture compositions are described in Table 1. The retention times of each compound and its corresponding absorption spectra are shown in Table 3. The data shown in this table is associated with Figure 5.

Table S5. Relative abundance, in percentages, of the representative compounds from the extracted phenolic acids.

Sample ID	C1	C2	Total phenolic acids
M1	0.00	100.00	100
M2	0.00	100.00	100
M3	0.00	100.00	100
M4	0.00	0.00	0
M5	0.00	100.00	100
M6	0.00	100.00	100
M7	0.00	100.00	100
M8	67.46	32.54	100

M1-M8 solvent mixture compositions are described in Table 1. The retention times of each compound and its corresponding absorption spectra are shown in Table 3.

Table S6. Correlation matrix (Pearson's *r* coefficients) between variables obtained from spectrophotometric analysis (TPC, TFC, FRAP, DPPH, ORAC) and chromatographic profiles (flavonoid related compounds). Significant correlations are indicated with * ($p < 0.05$) and ** ($p < 0.01$).

	TPC	TFC	FRAP	DPPH	ORAC	C3	C5	C8	C10	C12	C14	C15	C16	C18	C21
**TPC**	1														
**TFC**	0.957**	1													
**FRAP**	-0.214	-0.297	1												
**DPPH**	-0.310	-0.369	0.853**	1											
**ORAC**	-0.215	-0.298	0.989**	0.882**	1										
**C3**	-0.222	-0.307	0.989**	0.878**	1.000**	1									
**C4**	-0.210	-0.296	0.989**	0.884**	1.000**	1.000**									
**C5**	-0.219	-0.303	0.989**	0.882**	1.000**	1.000**	1								
**C6**	-0.218	-0.300	0.989**	0.882**	1.000**	1.000**	1.000**								
**C7**	-0.218	-0.300	0.989**	0.882**	1.000**	1.000**	1.000**								
**C8**	-0.181	-0.282	0.984**	0.888**	0.996**	0.996**	0.996**	1							
**C9**	0.214	0.037	0.024	0.178	0.034	0.044	0.037	0.115							
**C10**	-0.211	-0.295	0.989**	0.882**	1.000**	1.000**	1.000**	0.996**	1						
**C11**	-0.218	-0.300	0.989**	0.882**	1.000**	1.000**	1.000**	0.996**	1.000**						
**C12**	-0.212	-0.295	0.989**	0.882**	1.000**	1.000**	1.000**	0.996**	1.000**	1					
**C13**	-0.218	-0.300	0.989**	0.882**	1.000**	1.000**	1.000**	0.996**	1.000**	1.000**					
**C14**	-0.219	-0.302	0.989**	0.880**	1.000**	1.000**	1.000**	0.996**	1.000**	1.000**	1				
**C15**	-0.197	-0.278	0.988**	0.888**	0.999**	0.998**	0.998**	0.996**	0.999**	0.999**	0.999**	1			
**C16**	-0.218	-0.302	0.989**	0.882**	1.000**	1.000**	1.000**	0.996**	1.000**	1.000**	1.000**	0.999**	1		
**C17**	-0.278	-0.197	0.394	0.365	0.402	0.396	0.403	0.366	0.398	0.401	0.403	0.386	0.399		
**C18**	-0.218	-0.300	0.989**	0.881**	1.000**	1.000**	1.000**	0.996**	1.000**	1.000**	1.000**	0.998**	1.000**	1	
**C19**	-0.218	-0.300	0.989**	0.882**	1.000**	1.000**	1.000**	0.996**	1.000**	1.000**	1.000**	0.999**	1.000**	1.000**	
**C20**	-0.218	-0.300	0.989**	0.882**	1.000**	1.000**	1.000**	0.996**	1.000**	1.000**	1.000**	0.999**	1.000**	1.000**	
**C21**	0.502*	0.558**	-0.268	-0.008	-0.254	-0.261	-0.259	-0.232	-0.251	-0.251	-0.259	-0.219	-0.255	-0.258	1

Table S7. Correlation matrix (Pearson's *r* coefficients) between variables obtained from spectrophotometric analysis (TPC, TFC, FRAP, DPPH, ORAC) and chromatographic profiles (phenolic acids and anthocyanins related compounds). Significant correlations are indicated with * ($p < 0.05$) and ** ($p < 0.01$).

	TPC	TFC	FRAP	DPPH	ORAC	C1	C2	C4	C6	C7	C9	C11	C13	C17	C19	C20
**TPC**	1															
**TFC**	0.957**	1														
**FRAP**	-0.214	-0.297	1													
**DPPH**	-0.310	-0.369	0.853**	1												
**ORAC**	-0.215	-0.298	0.989**	0.882**	1											
**C1**	-0.218	-0.300	0.989**	0.882**	1.000**	1										
**C2**	-0.216	-0.300	0.988**	0.887**	1.000**	1.000**	1									
**C4**	-0.210	-0.296	0.989**	0.884**	1.000**	1.000**	1.000**	1								
**C6**	-0.218	-0.300	0.989**	0.882**	1.000**	1.000**	1.000**	1.000**	1							
**C7**	-0.218	-0.300	0.989**	0.882**	1.000**	1.000**	1.000**	1.000**	1.000**	1						
**C9**	0.214	0.037	0.024	0.178	0.034	0.033	0.045	0.051	0.033	0.033	1					
**C11**	-0.218	-0.300	0.989**	0.882**	1.000**	1.000**	1.000**	1.000**	1.000**	1.000**	0.033	1				
**C13**	-0.218	-0.300	0.989**	0.882**	1.000**	1.000**	1.000**	1.000**	1.000**	1.000**	0.033	1.000**	1			
**C17**	-0.278	-0.197	0.394	0.365	0.402	0.404	0.385	0.391	0.404	0.404	-0.379	0.404	0.404	1		
**C19**	-0.218	-0.300	0.989**	0.882**	1.000**	1.000**	1.000**	1.000**	1.000**	1.000**	0.033	1.000**	1.000**	0.404	1	
**C20**	-0.218	-0.300	0.989**	0.882**	1.000**	1.000**	1.000**	1.000**	1.000**	1.000**	0.033	1.000**	1.000**	0.404	1.000**	1

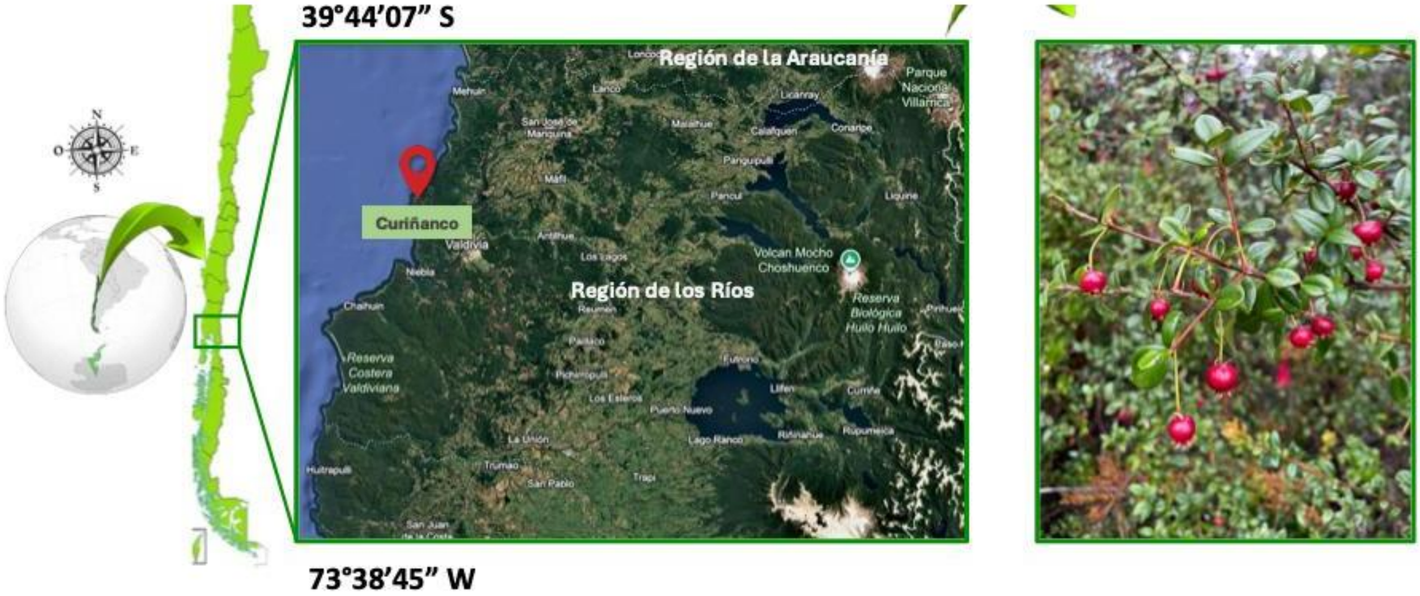

Figure S1. *Ugni molinae* fruits. The maps shows the place of collection of the ripe fruits.

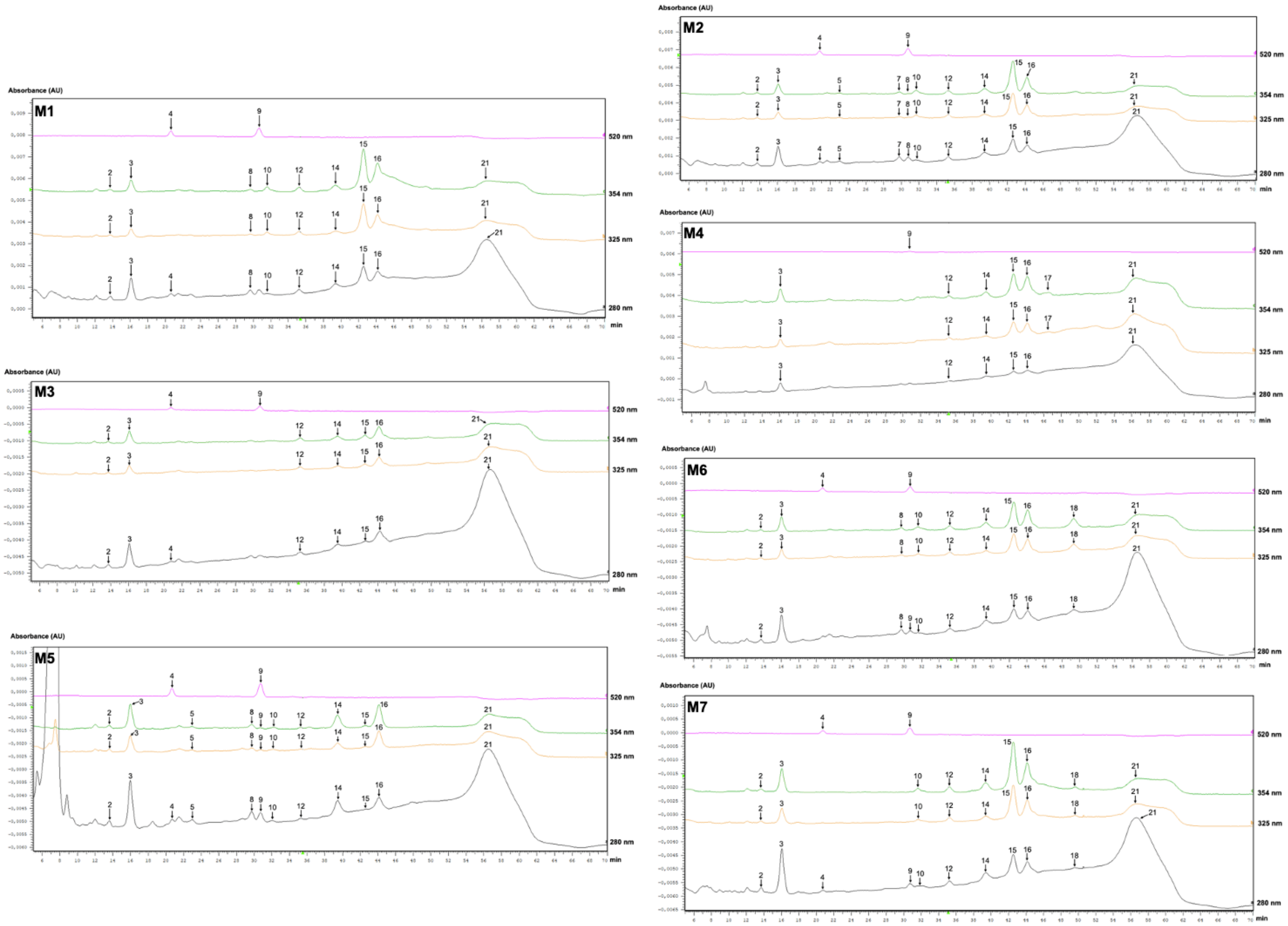

Figure S2. HPLC-DAD chromatograms of the extracts obtained at 280, 325, 354, and 520 nm from *U. molinae* fruits using NaDES as extraction solvents. M1-M7 solvent mixture composition are described in Table 1. The numbers of each peak are the same as Table 3.
